# Obstructive Sleep Apnea as a Risk Factor for Pneumonia Among Patients Infected SARS-CoV-2: A Propensity Score-Matching Analysis

**DOI:** 10.7759/cureus.93320

**Published:** 2025-09-26

**Authors:** Yanmei Cao, Yiming Zhao, Xinghua Shen, Yulin Kong, Jianping Zhang

**Affiliations:** 1 School of Public Health, Suzhou Medical College of Soochow University, Suzhou, CHN; 2 Department of Occupational Medicine, The Fifth People’s Hospital of Suzhou, Suzhou, CHN; 3 Department of Tuberculosis Medicine, The Fifth People’s Hospital of Suzhou, Suzhou, CHN

**Keywords:** chest computed tomography (ct), covid-19 pneumonia, obstructive sleep apnea (osa), propensity score matching (psm), sars-cov-2

## Abstract

Background

Obstructive sleep apnea (OSA) and coronavirus disease 2019 (COVID-19) share key pathophysiological features, including intermittent hypoxemia, systemic inflammation, and endothelial dysfunction, which may worsen clinical outcomes. However, whether OSA independently increases the risk of COVID-19 pneumonia remains unclear. This study aimed to assess the association between OSA and the risk of COVID-19 pneumonia in patients infected with severe acute respiratory syndrome coronavirus 2 (SARS-CoV-2).

Methodology

In this single-center, cross-sectional study, 136 hospitalized patients with confirmed SARS-CoV-2 infection were evaluated. OSA was diagnosed using home sleep apnea testing (HSAT), defined by an apnea-hypopnea index (AHI)≥five events/hour. Propensity score matching (PSM) was performed at a 1:1 ratio based on age, sex, body mass index (BMI), smoking history, and hypertension. Multivariate logistic regression was used to identify independent risk factors for COVID-19 pneumonia.

Results

The overall prevalence of OSA among patients infected with SARS-CoV-2 was 36.76% (50/136). After PSM, 32 patients with OSA were matched to 32 controls without OSA. The prevalence of pneumonia-related abnormalities on chest computed tomography (CT), including patchy or ground-glass opacities, was significantly higher in the OSA group compared to the non-OSA group. Furthermore, patients with OSA exhibited more severe nocturnal hypoxemia, as reflected by elevated oxygen desaturation index (ODI) and increased total sleep time with oxygen saturation below 90% (TST90). Multivariate logistic regression analysis identified age ≥60 years (odds ratio (OR)=12.05, *p*=0.004) and the presence of OSA (OR=4.56, *p*=0.010) as independent risk factors of COVID-19 pneumonia.

Conclusions

OSA was common among patients infected with SARS-CoV-2 and was independently associated with an increased risk of developing COVID-19 pneumonia, even after adjustment for major confounders. Patients with OSA exhibited more frequent radiological evidence of pneumonia and worse sleep-related respiratory parameters.

## Introduction

Since the emergence of severe acute respiratory syndrome coronavirus 2 (SARS-CoV-2) in late 2019, the global outbreak of coronavirus disease 2019 (COVID-19) has caused substantial morbidity and mortality. Although the initial pandemic wave has subsided, intermittent epidemic resurgences continue to occur worldwide. Most individuals infected with SARS-CoV-2 experience mild symptoms or remain asymptomatic. However, a subset of patients progresses to viral pneumonia, often accompanied by varying degrees of respiratory dysfunction. In severe cases, the condition can become life-threatening. Therefore, identifying the key risk factors for disease progression remains critical for an early intervention and effective clinical management.

Obstructive sleep apnea (OSA) is characterized by recurrent upper airway collapse during sleep, resulting in intermittent hypoxemia, activation of the sympathetic nervous system, and systemic inflammation [1]. It is a common but often underdiagnosed sleep-related breathing disorder. In addition to established risk factors such as advanced age, obesity, hypertension, and cardiovascular disease [2-5], recent studies suggest that OSA may also influence the development and outcomes of COVID-19 [6,7]. OSA often coexists with obesity, hypertension, and insulin resistance. These metabolic and cardiovascular comorbidities are also recognized as high risk factors for COVID-19 severity [3,8]. Previous research has proposed that OSA may worsen viral pneumonia through several mechanisms. These include worsening hypoxia, promoting inflammation and damaging vascular endothelial function [9,10]. Given the substantial pathophysiological overlap between OSA and COVID-19, including chronic low-grade inflammation, endothelial dysfunction, and immune dysregulation, their potential synergistic effects have drawn increasing clinical attention [11-13].

Although several studies have explored the association between OSA and adverse outcomes in COVID-19 patients [14,15], research investigating the prevalence of OSA among individuals infected with SARS-CoV-2 and its potential role as an independent risk factor for COVID-19 pneumonia remain limited. Furthermore, because OSA often coexists with other risk factors such as advanced age and obesity, inadequate adjustment for confounding variables in previous studies may have compromised the validity and interpretability of their findings [8]. To address this issue, we conducted a single-center, cross-sectional study using home sleep apnea testing (HSAT) and propensity score matching (PSM) to investigate the association between OSA and COVID-19 pneumonia.

## Materials and methods

Study subjects

This was a single-center, cross-sectional study conducted at the Fifth People’s Hospital of Suzhou. Patients hospitalized with SARS-CoV-2 infection between February 13, 2022, and April 30, 2022, were enrolled in the study. The diagnosis of COVID-19 was based on the Diagnosis and Treatment Protocol for Novel Coronavirus Pneumonia (Trial Version 9) issued by the National Health Commission of China [16]. The inclusion criteria were: (1) stable dyspnea, defined as symptoms persisting for ≥four weeks with minimal fluctuation, characterized by a variation of no more than one grade in resting modified Medical Research Council (mMRC) dyspnea scale and the absence of paroxysmal nocturnal dyspnea episodes [17] and (2) age >16 years. Exclusion criteria were as follows: (1) unstable dyspnea, defined as progressively worsening symptoms within a four-week period, accompanied by an increase of ≥two grades on the mMRC dyspnea scale and the onset of paroxysmal nocturnal dyspnea [17] and (2) previous diagnosis of OSA.

Data collection

Clinical data were obtained from two sources. Retrospective data from medical records included age, sex, body mass index (BMI), smoking history, vaccination status, time to viral nucleic acid negativity, and comorbidities (hypertension, diabetes mellitus, and malignancy). C-reactive protein (CRP) and procalcitonin (PCT) results from routine clinical testing, measured using a Jet-iStar 3000 fully automated immunoassay analyzer (Joinstar Biomedical Technology Co., Ltd., Hangzhou, China). Prospective assessments conducted for this study included neck, waist, and hip circumference, chest computed tomography (CT) scans (Brightspeed Elite 16-slice spiral CT scanner; GE Healthcare, Chicago, IL, USA), pulmonary function testing (POWERbreathe K5 pulmonary function training device; POWERbreathe Technologies Ltd, Birmingham, UK), and interleukin-6 (IL-6) analysis (NovoCyte D2060R flow cytometry; ACEA Biosciences Inc., San Diego, CA, USA).

Home sleep apnea testing

Nocturnal sleep apnea was assessed using a Nox-T3 portable monitoring device (Nox Medical, Reykjavík, Iceland). The monitored parameters included nasal airflow pressure, snoring, chest and abdominal movements, pulse rate, peripheral oxygen saturation (SpO_2_), body movements, and sleep position. When using a threshold of apnea-hypopnea index (AHI) ≥five events/hour, the Nox-T3 portable monitor demonstrated a sensitivity of 95%, specificity of 69%, positive predictive value of 94%, and negative predictive value of 75%, in comparison to polysomnography (PSG) [18]. Trained medical professionals administered the device to hospitalized patients. Sleep data were considered valid only when the monitoring duration exceeded three hours. Obstructive sleep apnea (OSA) was diagnosed when the AHI was ≥five events/ hour.

Assessment using questionnaires

All patients completed the Generalized Anxiety Disorder-7 (GAD-7) questionnaire and Patient Health Questionnaire-9 (PHQ-9). The GAD-7 evaluates anxiety symptoms and comprises seven items, with total scores ranging from 0 to 21; higher scores reflect more severe anxiety [19]. The PHQ-9 evaluates depressive symptoms and comprises nine items, with total scores ranging from 0 to 27; higher scores indicate more severe depression [20]. The GAD-7 and PHQ-9 were used under the terms of the open access license from Pfizer Inc., with proper attribution to the original authors.

Statistical analysis

Continuous variables with a normal distribution were presented as mean±standard deviation (mean±SD), while those with a non-normal distribution were presented as median and interquartile range (median (P25, P75)). Comparisons of normally distributed continuous variables between groups were performed using Student’s t-test, while non-normally distributed variables were analyzed using the Wilcoxon two-sample test. Categorical variables were presented as numbers and percentages (n (%)). Between-group comparisons for categorical variables were conducted using the chi-square test or Fisher’s exact test, as appropriate. In univariate analysis, variables with *p*-values <0.1 were included in the multivariate logistic regression model to identify the potential risk factors associated with COVID-19 pneumonia. The data were statistically analyzed using Statistical Analysis System (SAS) version 9.3 (SAS Institute Inc., Cary, NC, USA). A two-sided *p*-value <0.05 was considered statistically significant.

## Results

Baseline characteristics of the study population

A total of 136 patients infected with SARS-CoV-2 were included in this study, among whom 21 (15.44%) were diagnosed with COVID-19 pneumonia. Within the study population, 79 (58.09%) were men and 57 (41.91%) were women, and their age ranged from 16 to 73 years. Based on HSAT results, the overall prevalence of OSA was 36.76% (50/136). Compared with the non-OSA group, patients in the OSA group had a significantly higher median age and mean BMI. The proportion of smokers was also significantly greater in the OSA group. Additionally, neck, waist, and hip circumferences were significantly larger in the OSA group. The prevalence of hypertension was also significantly higher among patients with OSA. Radiologically, among those diagnosed with COVID-19 pneumonia, patchy or ground-glass opacities on chest CT were observed more frequently in the OSA group than in the non-OSA group. In terms of sleep respiratory parameters, the OSA group showed significantly higher apnea and hypopnea, as well as elevated total sleep time with oxygen saturation <90% (TST90) and oxygen desaturation index (ODI). Conversely, both the mean and lowest resting room air pulse oximetry (SpO_2_) were significantly higher in the non-OSA group. There were no statistically significant differences between the two groups in GAD-7 and PHQ-9 scores. Similarly, no significant differences were observed in pulmonary function tests or inflammatory markers between the two groups. These baseline findings suggested that patients with OSA tended to have more adverse demographic and clinical characteristics, warranting further adjustment for potential confounders. The baseline characteristics of the study population are summarized in Table 1.

**Table 1:** Basic characteristics of the study population BMI Body mass index; GAD-7: Generalized anxiety disorder-7 Scale; PHQ-9: Patient Health Questionnaire-9 scale;  SpO_2_: Resting room air pulse oximetry;  TST90: total sleep time with oxygen saturation <90%; ODI: oxygen desaturation index; CT: computed tomography; FVC: function vital capacity; FEV1: forced expiratory volume in one second; CRP: C-reactive protein; PCT: procalcitonin; IL-6: interleukin-6.

Variable	All (N=136)	non-OSA (N=86)	OSA (N=50)	t/Z/ χ^2^	*p* value
Age (years)	34.00 (28.50,43.50)	33.00 (26.00,39.00)	38.50 (32.00,48.00)	Z=2.705	0.007
Sex					
Male	79 (58.09)	39 (45.35)	40 (80.00)	χ^2^=15.593	＜0.001
Female	57 (41.91)	47 (54.65)	10 (20.00)		
BMI (kg/m^2^)	24.14 ± 3.99	23.21 ± 3.99	25.73 ± 3.49	t=4.194	＜0.001
Smoking	23 (16.91)	10 (11.63)	13 (26.00)	χ^2^=4.648	0.031
Vaccine	125 (91.91)	79 (91.86)	46 (92.00)	NA	1
Negative conversion time (days)	8.00 (6.00, 10.00)	8.00 (6.00, 10.00)	9.00 (6.00, 12.00)	Z=1.751	0.08
Neck circumference (cm)	37.00 (34.00, 39.00)	36.00 (33.00, 38.00)	39.00 (37.00, 41.00)	Z=5.113	＜0.001
Waist circumference (cm)	88.00 (78.00, 94.00)	84.00 (74.00, 90.00)	94.00 (89.00, 99.00)	Z=5.347	＜0.001
Hip circumference (cm)	97.50 ± 7.29	95.95 ± 7.76	100.12 ± 5.58	t=4.035	＜0.001
Comorbidities					
Hypertension	11 (8.09)	3 (3.49)	8 (16.00)	NA	0.018
Diabetes	3 (2.21)	2 (2.33)	1 (2.00)	NA	1
Tumors	4 (2.94)	4 (4.65)	0 (0.00)	NA	0.296
Questionnaires					
GAD-7	1.00 (0.00, 4.00)	1.00 (0.00, 4.00)	1.00 (0.00, 5.00)	Z=-0.252	0.801
PHQ-9	2.00 (0.00, 5.00)	2.50 (0.00, 5.00)	2.00 (0.00, 5.00)	Z=-0.382	0.702
Sleep parameters					
Apnea (events/hour)	2.25 (0.90, 5.50)	1.30 (0.50, 2.60)	8.50 (3.80, 15.70)	Z=7.864	＜0.001
Hypopnea (events/hour)	0.15 (0.00, 1.45)	0.00 (0.00, 0.30)	2.65 (0.10, 8.30)	Z=5.987	＜0.001
Mean SpO_2_ (%)	95.21 ± 1.58	95.63 ± 1.35	94.49 ± 1.69	t=-4.141	＜0.001
Lowest SpO_2_ (%)	86.50 ± 6.17	88.19 ± 4.42	83.60 ± 7.57	t=-4.449	＜0.001
TST90 (%)	0.00 (0.00, 1.65)	0.00 (0.00, 1.00)	0.75 (0.00, 2.00)	Z=3.782	＜0.001
ODI (events/hour)	1.55 (0.60, 5.65)	1.00 (0.40, 1.90)	7.25 (2.50, 15.20)	Z = 6.901	＜0.001
Chest CT imaging					
Patchy/ground-glass opacity	21 (15.44)	6 (6.98)	15 (30.00)	χ^2^=12.836	＜0.001
Normal	115 (84.56)	80 (93.02)	35 (70.00)		
Pulmonary function test					
FVC (% predicted)	70.00 (62.00, 79.00)	70.00 (62.00, 80.00)	69.50 (61.00, 79.00)	Z=-0.514	0.608
FEV1 (% predicted)	64.00 (45.00, 77.00)	62.00 (44.00, 74.00)	66.50 (59.00, 80.00)	Z=2.634	0.308
FEV1/FVC	107.00 (97.00, 115.00)	106.00 (97.00, 114.00)	108.50 (97.00, 116.00)	Z=0.696	0.486
VC	69.40 ± 12.01	70.06 ± 11.72	68.39 ± 12.50	t=0.731	0.466
Inflammatory markers					
CRP (mg/L)	2.95 (0.90, 8.57)	2.31 (0.60, 7.93)	3.18 (1.80, 10.60)	Z=1.818	0.069
PCT (ng/ml)	0.10 (0.10, 0.20)	0.10 (0.07, 0.20)	0.11 (0.10, 0.21)	Z=1.075	0.282
IL-6 (pg/ml)	10.35 (9.10, 10.70)	10.40 (9.20, 10.80)	10.20 (9.10, 10.70)	Z=-0.699	0.484

Propensity score matching analysis

To minimize potential confounding, PSM was conducted at a 1:1 ratio, matching 32 patients with OSA to 32 patients without OSA. The matching variables included sex, age, BMI, smoking history, and history of hypertension. After matching, the baseline differences in these variables were eliminated, achieving a balance between the two groups. Following PSM, the incidence of patchy or ground-glass opacities on chest CT remained significantly higher in the OSA group than in the non-OSA group. In terms of sleep respiratory parameters, the OSA group showed significantly higher apnea, hypopnea, TST90, and ODI than the non-OSA group. In contrast, the lowest SpO_2_ was significantly higher in the non-OSA group. These post-matching findings indicate that OSA itself, independent of major confounding factors, may contribute to the radiological and respiratory differences observed between groups. Detailed results are summarized in Table 2.

**Table 2:** Propensity Score Matching Patients with and without OSA BMI: Body mass index; GAD-7: Generalized anxiety disorder-7 scale; PHQ-9: Patient health questionnaire-9 scale;  SpO_2_: resting room air pulse oximetry; TST90: total sleep time with oxygen saturation <90%; ODI: oxygen desaturation index; CT: computed tomography; FVC: function vital capacity; FEV1: forced expiratory volume in 1 second; CRP: C-reactive protein; PCT: Procalcitonin; IL-6: interleukin-6.

Variable	Non-OSA (N=32)	OSA (N=32)	Z/ χ^2^	*p* value
Age (years)	36.00 (32.00, 44.50)	38.00 (30.00, 45.00)	Z=0.06	0.952
Sex				
Male	21 (65.63)	25 (78.13)	χ^2^=1.237	0.266
Female	11 (34.38)	7 (21.88)		
BMI (kg/m^2^)	24.75 (22.80, 28.35)	24.20 (22.85, 25.95)	Z=-0.638	0.523
Smoking	7 (21.88)	7 (21.88)	χ^2^=<0.001	1
Vaccine	29 (90.63)	29 (90.63)	NA	1
Negative conversion time (days)	8.00 (6.00, 10.00)	8.00 (6.00, 10.50)	Z=-0.257	0.7997
Neck circumference (cm)	38.50 (37.00, 39.00)	39.00 (35.50, 40.50)	Z=0.696	0.486
Waist circumference (cm)	90.00 (84.50, 94.50)	92.00 (87.50, 98.00)	Z=1.009	0.313
Hip circumference (cm)	97.50 (94.00, 102.50)	100.00 (96.00, 102.50)	Z=0.78	0.435
Hypertension	3 (9.38)	1 (3.13)	NA	0.613
GAD-7	2.00 (0.00, 5.00)	1.00 (0.00, 5.00)	Z=-0.795	0.427
PHQ-9	4.00 (0.00, 5.50)	2.00 (0.00, 7.00)	Z=-0.138	0.89
Sleep parameters				
Apnea (events/hour)	1.45 (0.45, 2.35)	8.60 (3.70, 16.20)	Z=5.265	<0.001
Hypopnea (events/hour)	0.00 (0.00, 0.20)	3.20 (0.30, 8.25)	Z=4.81	<0.001
Mean SpO_2_ (%)	95.40 (94.25, 96.00)	95.05 (93.80, 95.35)	Z=-1.566	0.117
Lowest SpO_2_ (%)	89.00 (86.50, 91.00)	85.00 (80.50, 90.00)	Z=-3.182	0.001
TST90 (%)	0.00 (0.00, 1.00)	0.50 (0.00, 3.45)	Z=2.818	0.005
ODI (events/hour)	1.00 (0.40, 2.00)	8.10 (2.55, 15.85)	Z=4.755	<0.001
Chest CT imaging				0.023
Patchy/ground-glass opacity	2 ( 6.25)	9 (28.13)	χ^2^=5.132	0.023
Normal	30 (93.55)	23 (71.88)		
Pulmonary function test				
FVC (% predicted)	72.00 (62.00, 78.00)	70.50 (62.00, 79.00)	Z=0.312	0.755
FEV1 (% predicted)	72.00 (46.00, 78.00)	71.50 (56.50, 79.00)	Z=-0.615	0.539
FEV1/FVC	107.50 (100.00, 112.00)	110.00 (100.50, 115.50)	Z=-0.832	0.405
VC	73.00 (64.00, 78.00)	67.50 (62.50, 79.00)	Z=0.463	0.643
Inflammatory markers				
CRP (mg/L)	2.58 (0.55, 6.40)	3.03 (1.01, 9.95)	Z=0.909	0.363
PCT (ng/ml)	0.13 (0.10, 0.21)	0.16 (0.10, 0.22)	Z=-0.683	0.494
IL-6 (pg/ml)	9.90 (8.10, 10.70)	10.00 (8.90, 10.70)	Z=0.042	0.967

Risk factors for COVID-19 pneumonia

To further explore the risk factors of COVID-19 pneumonia, multivariate logistic regression analysis identified age ≥60 years and the presence of OSA as independent risk factors for COVID-19 pneumonia among patients infected with SARS-CoV-2. Compared with patients aged <60 years, those aged ≥60 years had a significantly higher risk of developing COVID-19 pneumonia (OR=12.05, 95%CI: 2.23-64.98, *p*=0.004). Furthermore, patients with OSA had a significantly higher risk of developing COVID-19 pneumonia compared to those without OSA (OR=4.56, 95% CI: 1.44-14.42, *p*=0.010). Together, these findings consistently support OSA as a significant and independent predictor of COVID-19 pneumonia. Detailed results are presented in Table 3.

**Table 3:** Risk Factors for COVID-19 Pneumonia COVID-19: Coronavirus disease 2019; OSA: obstructive sleep apnea; OR: odds ratio; CI: confidence interval

Variable		OR (95%CI)	*p* value
Age	≥ 60 years vs. < 60 years	12.05 (2.23, 64.98)	0.004
Vaccine	≤ Two doses vs. three doses	3.40 (0.76, 15.15)	0.108
Hypertension	Yes vs. No	2.01 (0.36, 11.26)	0.428
OSA	Yes vs. No	4.56 (1.44, 14.42)	0.010

## Discussion

This study investigated the association between OSA and the COVID-19 pneumonia in patients infected with SARS-CoV-2. We found that OSA was highly prevalent in this population (36.76%) and is identified as an independent risk factor for COVID-19 pneumonia, even after adjusting for major confounders.

We observed a notably high prevalence of OSA in our study, consistent with previous reports suggesting that sleep-disordered breathing is often underdiagnosed in hospitalized populations. Evidence in this field has been evolving. A 2021 study reported OSA was significantly more common in hospitalized COVID-19 patients compared with non-hospitalized individuals, particularly among those who develop respiratory failure [21]. This cohort study demonstrated that, even after adjusting for BMI, hypertension, and diabetes, OSA independently increased the risk of hospitalization (OR 1.65) and respiratory failure (OR 1.98) [21]. More recently, a study that enrolled 125 hospitalized COVID-19 patients between September 2020 and April 2021 found that 82% were diagnosed with OSA, and both the AHI and ODI were significant predictors of the need for advanced respiratory support [22]. Most notably, a 2024 systematic review and meta-analysis including 48 studies and over 8.6 million individuals confirmed that OSA is independently associated with increased risks of COVID-19 infection, hospitalization, mortality, and long COVID [23]. Taken together, these findings - including ours - suggested that OSA itself, beyond traditional risk factors, may play an independent role in exacerbating COVID-19 severity. Our study, using rigorous propensity score matching, further strengthens this evidence by showing that OSA remains significantly associated with COVID-19 pneumonia after controlling for key confounders.

Patients in the OSA group were generally older and had higher BMI, as well as greater neck, waist, and hip circumferences. They also showed higher rates of smoking and hypertension. These factors are recognized as established risk factors and common comorbidities for both OSA and COVID-19. These observations align with previous studies and highlight the necessity of using PSM to reduce potential confounding. Notably, even after adjusting for key variables known to affect COVID-19 severity, including age, BMI, smoking history, and hypertension, OSA remained significantly associated with the development of COVID-19 pneumonia. This strengthens the validity of the observed relationship. Following PSM, patients with OSA continued to show a higher prevalence of patchy or ground-glass opacities on chest CT, along with significantly worse sleep-related respiratory parameters. These findings further support a potential independent pathogenic role of OSA in the progression of COVID-19 pneumonia. The results underscore the importance of increased clinical awareness of undiagnosed OSA in COVID-19 patients, particularly in high-risk groups such as the elderly and those with obesity or hypertension. Early identification of OSA in these populations may improve risk stratification and inform targeted management strategies, including enhanced respiratory monitoring and timely initiation of continuous positive airway pressure (CPAP) therapy during acute infection, potentially improving clinical outcomes.

The observed association between OSA and COVID-19 pneumonia can be explained by several overlapping pathophysiological mechanisms. The key pathophysiological features of OSA, such as chronic intermittent hypoxia (CIH), oxidative stress, endothelial dysfunction, and systemic inflammation, are also implicated in the pathogenesis of COVID-19. Among these, CIH is considered a major driver of disease severity. Repetitive hypoxia-reoxygenation cycles activate hypoxia-inducible factor-1α (HIF-1α), leading to dysregulated expression of vascular endothelial growth factor (VEGF) and endothelial nitric oxide synthase (eNOS). This disruption impairs endothelial integrity, increases vascular permeability, and facilitates pulmonary vascular leakage, thereby promoting COVID-19-related pneumonia [24,25]. Moreover, CIH is strongly associated with excessive production of reactive oxygen species (ROS) and suppression of endogenous antioxidant defenses [26]. The resulting oxidative stress may act synergistically with SARS-CoV-2-induced oxidative injury, exacerbating damage to alveolar epithelial and vascular endothelial cells [27]. CIH and ROS exposure also activate the nuclear factor kappa B (NF-κB) signaling pathway, resulting in increased expression of pro-inflammatory cytokines, such as tumor necrosis factor-α (TNF-α), IL-6, and IL-1β [28]. This pre-existing inflammatory state may potentiate the COVID-19 cytokine storm, thereby amplifying pulmonary inflammation and injury. These mechanisms together provide a plausible biological explanation for why OSA patients in our study demonstrated a higher prevalence of patchy or ground-glass opacities on chest CT compared with non-OSA patients [29]. Radiological findings of diffuse alveolar damage may thus reflect the combined effects of SARS-CoV-2-induced inflammation and OSA-related hypoxia, oxidative stress, and endothelial dysfunction. Importantly, this also explains why the association between OSA and COVID-19 pneumonia persisted even after adjusting for traditional risk factors such as age, obesity, and hypertension.

Interestingly, no significant differences were observed between groups in inflammatory markers (C-reactive protein (CRP), procalcitonin (PCT), and interleukin-6 (IL-6)) or pulmonary function parameters. Several factors may explain these results. First, in the context of acute viral infection, these markers are influenced by multiple confounders, which may obscure subtle intergroup differences. Second, some patients may have been in the early phase of infection, before the onset of a robust systemic inflammatory response. Third, the relatively small sample size limited the power to detect modest differences. Finally, the pathogenic impact of OSA may be more localized, primarily affecting pulmonary structure and gas exchange, rather than systemic inflammation, which conventional markers may fail to capture. Future studies should include larger cohorts and a wider range of molecular and physiological indicators to better define the role of OSA in COVID-19 pneumonia progression.

This study has several limitations. First, it was a single-center retrospective analysis with a relatively small sample size, which may limit the generalizability of the findings. Second, although PSM was applied to reduce the major confounding factors, the potential impact of residual confounders cannot be entirely excluded. Third, matching further reduced the sample size, possibly lowering the statistical power to detect differences in some secondary outcomes. Finally, OSA diagnosis was based on portable sleep monitoring rather than polysomnography, the gold standard, which may have led to an underestimation of OSA severity.

## Conclusions

In conclusion, our study demonstrated that OSA was common among patients infected with SARS-CoV-2 and was independently associated with an increased risk of developing COVID-19 pneumonia, even after adjusting for major confounders. Patients with OSA exhibited more frequent radiological evidence of pneumonia and worse sleep-related respiratory parameters, suggesting a potential contributory role of OSA in COVID-19 progression. These findings highlight the importance of early recognition and management of OSA in SARS-CoV-2-infected individuals, particularly in high-risk groups such as the elderly and those with obesity or hypertension. Future multicenter studies with larger cohorts and polysomnography-based diagnosis are needed to validate these results and clarify the underlying mechanisms.
